# Supplementary material for: Long-term taxonomic and functional divergence from donor bacterial strains following fecal microbiota transplantation in immunocompromised patients
Source: PLoS One. 2017 Aug 21;12(8):e0182585. doi: 10.1371/journal.pone.0182585 (PMC5565110; doi:10.1371/journal.pone.0182585)
Supplement: S1 Table — Taxonomic abundances were calculated with the Kraken classifier using a custom database constructed from the NCBI Genbank and Refseq reference collections. Similarity is expressed for each subject to the appropriate donor sample, and for each time point in the donor 29 time series to the initial time point in that series. (DOCX) [file pone.0182585.s001.docx]

| Taxonomic Donor Similarity | Subject | Days From FMT/Initial Timepoint |
| --- | --- | --- |
| 0.08 | A | -1 |
| 0.4 | A | 6 |
| 0.29 | A | 14 |
| 0.58 | A | 16 |
| 0.51 | A | 21 |
| 0.3 | A | 408 |
| 0.04 | B | -1 |
| 0.74 | B | 6 |
| 0.63 | B | 8 |
| 0.56 | B | 13 |
| 0.68 | B | 20 |
| 0.24 | B | 384 |
| 1 | Donor | 0 |
| 0.62 | Donor | 33 |
| 0.54 | Donor | 35 |
| 0.46 | Donor | 37 |
| 0.79 | Donor | 40 |
| 0.54 | Donor | 41 |
| 0.52 | Donor | 42 |
| 0.68 | Donor | 232 |
| 0.22 | C | 456 |
| 0.41 | D | 179 |
| 0.53 | E | 448 |
| 0.41 | F | 410 |
