# Supplementary material for: Long-term taxonomic and functional divergence from donor bacterial strains following fecal microbiota transplantation in immunocompromised patients
Source: PLoS One. 2017 Aug 21;12(8):e0182585. doi: 10.1371/journal.pone.0182585 (PMC5565110; doi:10.1371/journal.pone.0182585)
Supplement: S2 Table — Gene family abundances were calculated by alignment to the UniRef50 database. Similarity is expressed for each subject to the appropriate donor sample, and for each time point in the donor 29 time series to the initial time point in that series. (DOCX) [file pone.0182585.s002.docx]

| Functional Donor Similarity | Subject | Days From FMT/Initial Timepoint |
| --- | --- | --- |
| 0.53 | A | -1 |
| 0.97 | A | 6 |
| 0.96 | A | 14 |
| 0.97 | A | 16 |
| 0.98 | A | 21 |
| 0.93 | A | 408 |
| 0.79 | B | -1 |
| 0.88 | B | 6 |
| 0.9 | B | 8 |
| 0.93 | B | 13 |
| 0.92 | B | 20 |
| 0.72 | B | 384 |
| 1 | Donor | 0 |
| 0.98 | Donor | 33 |
| 0.98 | Donor | 35 |
| 0.98 | Donor | 37 |
| 0.99 | Donor | 40 |
| 0.99 | Donor | 41 |
| 0.98 | Donor | 42 |
| 0.99 | Donor | 232 |
| 0.81 | C | 456 |
| 0.87 | D | 179 |
| 0.72 | E | 448 |
| 0.89 | F | 410 |
