# Supplementary material for: Long-term taxonomic and functional divergence from donor bacterial strains following fecal microbiota transplantation in immunocompromised patients
Source: PLoS One. 2017 Aug 21;12(8):e0182585. doi: 10.1371/journal.pone.0182585 (PMC5565110; doi:10.1371/journal.pone.0182585)
Supplement: S3 Table — Antibiotic resistance gene abundances were measured by alignment to the CARD antibiotic resistance database and normalizing by per-sample coverage. (DOCX) [file pone.0182585.s003.docx]

| Days From FMT/Initial Timepoint | Chao Donor Similarity |
| --- | --- |
| -1 | 0.73 |
| 6 | 0.96 |
| 14 | 0.93 |
| 16 | 0.97 |
| 21 | 0.98 |
| 408 | 0.9 |
