# Supplementary material for: Long-term taxonomic and functional divergence from donor bacterial strains following fecal microbiota transplantation in immunocompromised patients
Source: PLoS One. 2017 Aug 21;12(8):e0182585. doi: 10.1371/journal.pone.0182585 (PMC5565110; doi:10.1371/journal.pone.0182585)
Supplement: S4 Table — These read files have been submitted to NCBI SRA under BioProject accession No. PRJNA349197. (DOCX) [file pone.0182585.s004.docx]

| Raw Read Count | Subject | Days From FMT/Initial Timepoint |
| --- | --- | --- |
| 37122926 | A | -1 |
| 58219286 | A | 6 |
| 50900570 | A | 14 |
| 46955542 | A | 16 |
| 53273498 | A | 21 |
| 36170964 | A | 408 |
| 39909812 | B | -1 |
| 32572758 | B | 6 |
| 39558652 | B | 8 |
| 15286048 | B | 13 |
| 25577246 | B | 20 |
| 47893524 | B | 384 |
| 28954438 | Donor 29 | 0 |
| 20136302 | Donor 29 | 33 |
| 15817486 | Donor 29 | 35 |
| 14314808 | Donor 29 | 37 |
| 31065310 | Donor 29 | 40 |
| 16549506 | Donor 29 | 41 |
| 15263036 | Donor 29 | 42 |
| 36717190 | Donor 29 | 232 |
| 40918176 | C | 456 |
| 17821536 | D | 179 |
| 21676804 | E | 448 |
| 29516870 | F | 410 |
| 43865284 | Donor 28 | NA |
| 35024920 | Donor 32 | NA |
