# Supplementary figures and images for: Long-term taxonomic and functional divergence from donor bacterial strains following fecal microbiota transplantation in immunocompromised patients
Source: PLoS One. 2017 Aug 21;12(8):e0182585. doi: 10.1371/journal.pone.0182585 (PMC5565110; doi:10.1371/journal.pone.0182585)

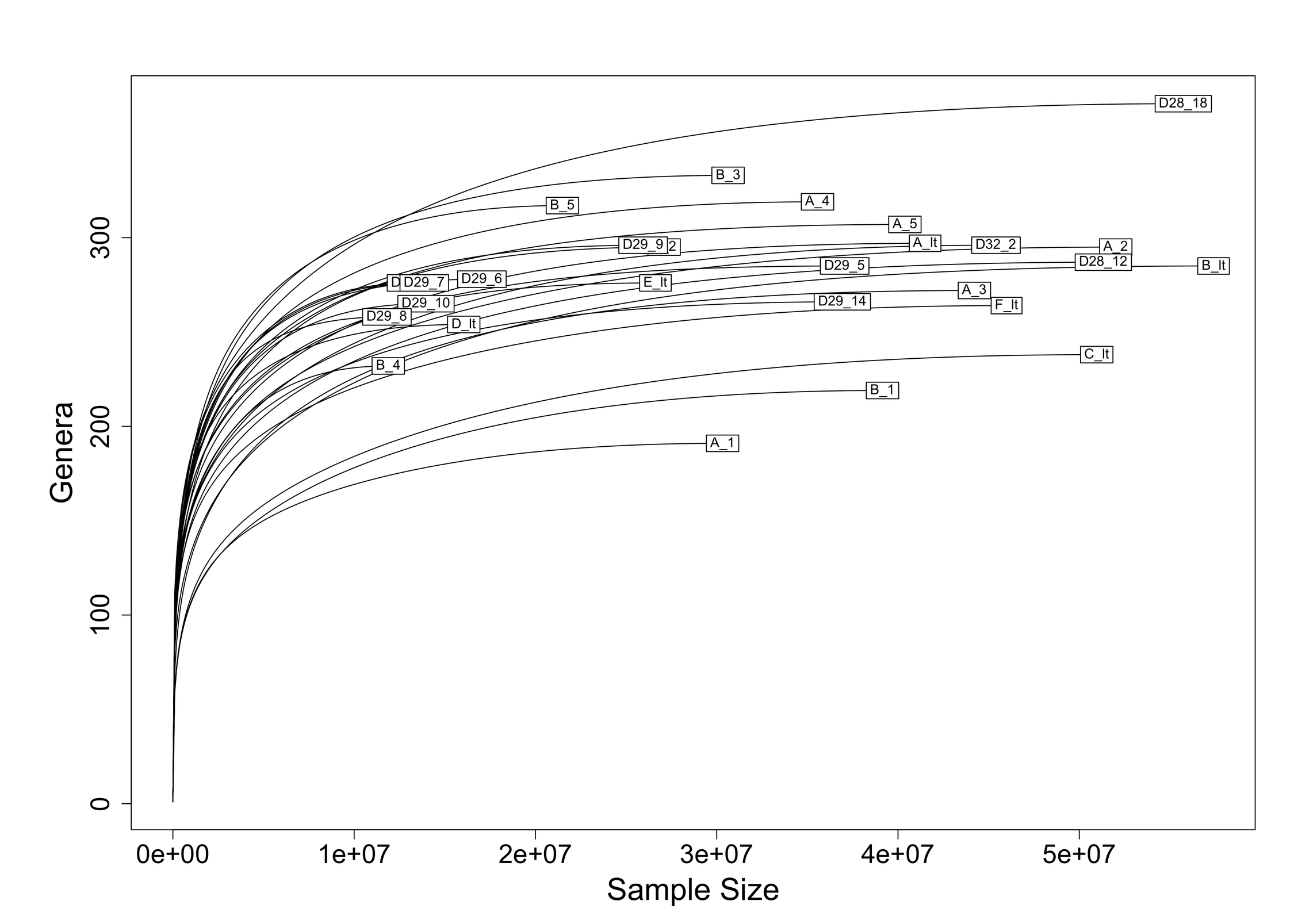

Supplement: S1 Fig — Rarefaction curves were calculated using the vegan library(Oksanen et al, 2007) in the R statistical computing language(Ihaka & Gentleman, 1996). Curves represent repeated random subsamplings of genus-level taxonomic calls taken at increasing sample sizes. Singleton calls were omitted from this analysis. We assess sufficient sampling when a curve has transitioned from the vertical phase to an asymptotic phase. Samples are labeled in the form “Subject_Timepoint”, with ‘lt’ identifying long-term timepoints and donor samples prefixed with the letter D. Timepoints are numbered in chronological order for each subject. Note that labels D29_11 and D29_7 are coincident, as are D29_9 and B_2. (TIF) [file pone.0182585.s005.tif]
